# Is Darwinism a Dying Faith?

**Published:** 1894-02-24

**Authors:** 


### The Hospital

An Institutional Journal of

The Medical Sciences and Hospital Administration, with

AN EXTRA NURSING SUPPLEMENT.

Vol. XV.—No. 387.] [Feb. 24, 1894.

### Is Darwinism a Dying Faith?

Dr. Hutchinson Stirling, first Gifford Lecturer at the University of Edinburgh, is a man of much capacity for reading, and of some power of thinking: he thinks that the late Mr. Darwin is to a considerable extent "played out." Mr. Alfred Russell Wallace who, in addition to being a wide reader, is a voluminous writer on biological subjects, is very angry indeed with Dr. Hutchinson Stirling for his "presumption." No less than six columns of *Nature* were devoted to the scourging of Dr. Stirling's shoulders by Mr. Wallace's pen a week or two ago. This, we suggest, is not the temper in which scientific subjects should be discussed. It divides the army of science into opposite camps, and substitutes personal ends for scientific simplicity of purpose. Moreover, it causes the enemies of science to blaspheme.

The "struggle for survival," which is one of the chief planks in the platform of the Darwinian position, is vigorously assailed by Dr. Hutchinson Stirling. There is no struggle, he says. On the contrary, there is a great deal of amicability throughout the vegetal and animal worlds; and a wide survey discovers a general condition, at least of truce, if not of peace. If the wolf does not dwell with the lamb, he, at any rate, manages to live on terms of friendship with his brother wolf; and if the leopard does not actually lie down with the kid, he is so very considerate to the antelope and many other varieties of herbivorous animal life in the primeval forest, that all sorts and conditions of those creatures manage to survive side by side with manifold varieties of the dreaded carnivora. The "survival of the fittest," which is the mere complement of the struggle for survival, is an abomination to Dr. Stirling; it is, he considers, stark nonsense. He pushes the doctrine to what he holds to be its logical and inevitable conclusion. If in the past the "fittest" alone had survived, then the unfit must gradually have become extinct; and the world, having been struggling for absolutely uncountable ages, the struggle must have come to an end by this time with the survival of a solitary unit of animal or vegetal life. The picture is decidedly striking; and the logic is plausible, if not convincing. Dr. Hutchinson Stirling is clearly not without some capacity for broad survey and philosophical analysis and synthesis. Let him continue his critical investigations by all means.

But the question still remains, Was Darwin wholly or mainly right in his leading positions? and the question has its practical as well as its academic side. It must, therefore, be systematically argued by competent thinkers in the interests of rational living and human development, as well as of scientific reputations. It is a question which cannot be profitably discussed by mere appeals to scientific authority, or even to philosophic plausibility. The appeal must constantly be pushed back upon facts; facts present and facts historical. The question to be asked is not which of several hypotheses is the more plausible, but which is the true? And in the answering of this question mere names must not only not be allowed to have undue weight, but they must be sacrificed without hesitation if they stand in the way of the progress of truth.

It is a fact not without significance that the very phrases "struggle for survival" and "survival of the fittest" at once "caught on," to use an expressive colloquialism, all over the civilised world, and in every class of society. They seemed to strike a simultaneous chord in all classes of human hearts and intellects which had been waiting for ages for the stroke of the master's hand. That is a fact which is worth thinking of in this controversy. For after all it is the human mind which establishes all science for us, and it is human experience which makes for us the candle of the highest illuminating power wherewith to explore the whole region of non-personal things in their openest or their most secret abodes. That there is a struggle in that external nature which is not ourselves we most of us think we can clearly see, and that there is a struggle within ourselves of our own personal forces against one another, and of our united forces against the external world, all of us who have any age and experience at all are absolutely sure. This, too, is a fact to be insisted upon. Man, being moral and intellectual as well as material, is in a very peculiar sense a cosmos, and it seems to be *a priori* exceedingly probable that what we find in him as a type we shall find in all external nature which is in any way associated with him.

Darwin looked for his facts more particularly in the non-human world, or in the human world in half-developed and very primitive conditions. In accumulating vast stores of facts of this order he rendered a service to science which has never been surpassed. Dr. Hutchinson Stirling contends against him that he was a mere compiler, and not a laboratory worker. If we were not anxious to avoid the "scolding" tone of Mr. Russell Wallace we should say that this is a ridiculous contention. Although Darwin was not a laboratory worker, he could have been one if he had possessed the quite unusual power of being able to be present in two places at once. His work was that of a wide observer in the great outside laboratory of nature, and of the philosophical system propounder in his study. That was surely work enough for one delicate man: and to complain of him that he was a mere compiler because he used the facts observed and recorded by other men, as well as those observed and recorded by himself, is at least disingenuous if it is not foolish. Darwin certainly had hypotheses of his own to maintain; he was a great theoriser. But this need only concern the world of to-day in so far as it is called upon to prove whether his hypotheses were true or not. When Dr. Stirling makes, as he does, a very serious effort to belittle Darwin in order thereby to diminish general confidence in his scientific capacity, we feel that he also may have an "axe to grind," and an axe which is not entirely to his credit as an honest searcher and expounder of scientific truth.

The great hypothesis of Darwin—that there is throughout nature a continuous struggle for survival and that the fittest are destined to survive—seems to us to express a real truth, and a truth as important as it is real. It is a very striking circumstance, and one which an open-eyed philosopher cannot but take account of, that the Hebrew Paul, who became the chief exponent of the Christian faith in its struggling days, should have had a profound appreciation of this very struggle. "We know," says he, "that the whole creation groaneth and agoniseth in pain together until now . . . waiting." There, nearly two thousand years ago, was a man who had modern eyes; and who had no scientific hypothesis to maintain. He was not a Greek theoriser nor a Roman poet-philosopher; he was neither a Democritus nor a Lucretius, but a teacher of religion. Let it be remembered in these scientific days to his honour, that he saw external nature, and his own inner nature too, with the clearness of a philosopher, and placed out indistinct and picturesque language for all time this reasoned conclusion—that there is throughout all those regions of the universe which come beneath the cognisance of man, a "struggle," an "agonising," and a "waiting"—an attempt in fact at the "survival of the fittest."

The survival of the fittest, it must be remembered, does not always, or perhaps ever, mean the survival of the merely strongest, or the merely selfishest. The very powerful or the excessively selfish man or animal often becomes acutely obnoxious to other men and animals; and his powerful selfishness may be the very cause of his destruction rather than of his survival. The wisest, not the cunningest, but the wisest, animal, the creature who has the finest appreciation of the principle of "live and let live," would appear to be the one most adapted for survival in a world where struggles, and battles, and alliances, and the seeking of help from all possible quarters, is the order of the day. In such a world those creatures which on the one hand make the least demands upon others, and on the other show the greatest capacity and willingness to render services to others, are surely the most fitted for survival; and, as a matter of fact, it seems undoubted that such are the creatures which survive. In our judgment the doctrine of the "survival of the fittest" is sound in philosophy; and the principle in fact and practice is not only discoverable but is of the highest service to the world.

